# Visfatin Promotes Wound Healing through the Activation of ERK1/2 and JNK1/2 Pathway

**DOI:** 10.3390/ijms19113642

**Published:** 2018-11-19

**Authors:** Byung-Cheol Lee, Jisun Song, Arim Lee, Daeho Cho, Tae Sung Kim

**Affiliations:** 1 Department of Life Sciences, College of Life Sciences and Biotechnology, Korea University, Seoul 136-701, Korea; microlbc@korea.ac.kr (B.-C.L.); wltjs529@hanmail.net (J.S.); arim323@korea.ac.kr (A.L.); 2 Institute of Convergence Science, Korea University, Seoul 136-701, Korea; cdhkor@korea.ac.kr

**Keywords:** visfatin, wound healing, VEGF, MAPK

## Abstract

Visfatin, a member of the adipokine family, plays an important role in many metabolic and stress responses. The mechanisms underlying the direct therapeutic effects of visfatin on wound healing have not been reported yet. In this study, we examined the effects of visfatin on wound healing in vitro and in vivo. Visfatin enhanced the proliferation and migration of human dermal fibroblasts (HDFs) and keratinocytes the expression of wound healing-related vascular endothelial growth factor (VEGF) in vitro and in vivo. Treatment of HDFs with visfatin induced activation of both extracellular signal-regulated kinases 1 and 2 (ERK1/2) and c-Jun N-terminal kinases 1 and 2 (JNK1/2) in a time-dependent manner. Inhibition of ERK1/2 and JNK1/2 led to a significant decrease in visfatin-induced proliferation and migration of HDFs. Importantly, blocking VEGF with its neutralizing antibodies suppressed the visfatin-induced proliferation and migration of HDFs and human keratinocytes, indicating that visfatin induces the proliferation and migration of HDFs and human keratinocytes via increased VEGF expression. Moreover, visfatin effectively improved wound repair in vivo, which was comparable to the wound healing activity of epidermal growth factor (EGF). Taken together, we demonstrate that visfatin promotes the proliferation and migration of HDFs and human keratinocytes by inducing VEGF expression and can be used as a potential novel therapeutic agent for wound healing.

## 1. Introduction

The three layers of skin contain the human dermal fibroblasts (HDFs), epidermal keratinocytes, immune cells, nerves, and intradermal adipocytes [1,2,3]. The constant communication between the different cellular constituents of various compartments of the skin and its extracellular matrix (ECM) plays a critical role in maintaining physiological homeostasis of the body [4,5,6]. The most essential resident cell of the dermis is HDF which is involved in wound healing through the production of ECM [4,5]. HDFs communicate with each other and with other cell types, including keratinocytes, immune cells, and adipocytes, thus playing a crucial role in regulating skin physiology and tissue repair [2,7]. Tissue repair immediately starts after the skin gets damaged. Wound repair is a tightly regulated complex process, which includes hemostasis, inflammation, proliferation, and re-epithelialization [8,9]. During wound healing, proliferation and migration of resident HDFs into the damaged area results in the recovery of skin wounds [10,11].

Visfatin, also known as pre-B cell colony-enhancing factor (PBEF) or nicotinamide phosphoribosyl-transferase (NAMPT), was originally cloned from activated peripheral blood lymphocytes in 1994, as a highly conserved intracellular and extracellular 52 kDa adipokine [12,13,14,15,16]. Visfatin is released from various cell types and tissues of higher organisms, including peripheral-blood monocytes, lymphocytes, dendritic cells, macrophages, adipose tissues, in particular visceral adipose tissue and is expressed in various malignant tumors, including colon, stomach, pancreas, liver, prostate, and breast cancers [13,15,17,18,19,20,21,22,23,24]. To date, two forms of visfatin have been identified: intracellular and extracellular. Intracellular visfatin plays a key role in maintaining the activity of nicotinamide adenine dinucleotide (NAD)-dependent enzymes and is implicated in the regulation of cellular metabolism. A recent study has reported that both intracellular and extracellular visfatin have been shown to be involved in tumor development [25]. Additionally, many studies have demonstrated that visfatin promotes angiogenesis by enhancing the proliferation, migration, formation of functional neovessels, and invasion of vascular endothelial cells in vitro and in vivo [26,27,28,29]. Further, visfatin has been shown to exhibit anti-apoptotic and proinflammatory properties [30]. Previous studies have reported the potential effects of visfatin on the proliferation and collagen synthesis in rat cardiac tissue and on induction of vascular smooth muscle cells [31].

Vascular endothelial cell growth factor (VEGF) is an angiogenic factor and the most important regulator of cell proliferation, migration, and permeabilization [32,33,34,35]. Many studies have reported that VEGF plays a critical role in wound healing through the induction of vascular permeability [32,36]. VEGF is produced in many cell types, including tumor cells and healthy cells, such as epidermal cells of the skin, including keratinocytes and fibroblasts [32,37,38]. A recent study has suggested that visfatin-induced proliferation of endothelial cells relies on the synthesis and secretion of VEGF [39], a key regulator of cell proliferation and angiogenesis. Moreover, visfatin has been shown to up-regulate the VEGF receptor 2, which mediates the angiogenic actions of VEGF [27,39]. Visfatin has been reported to regulate the expression of VEGF through activation of several pathways, including phosphatidylinositol 3-kinase (PI3K)/Akt, the mitogen-activated protein kinases (MAPK) extracellular signal-regulated kinases 1 and 2 (ERK1/2), and p38 signaling pathways [26,27,39,40,41].

Thus, we hypothesized that visfatin might induce wound healing through the induction of proliferation and migration. However, to date, there is poor evidence on the functions and direct effect of visfatin on wound healing in vitro and in vivo. In the present study, we aimed to evaluate the effect of visfatin on cell proliferation and migration.

## 2. Results

### 2.1. Visfatin Enhances the Proliferation and Migration of HDFs

**Figure 1:**
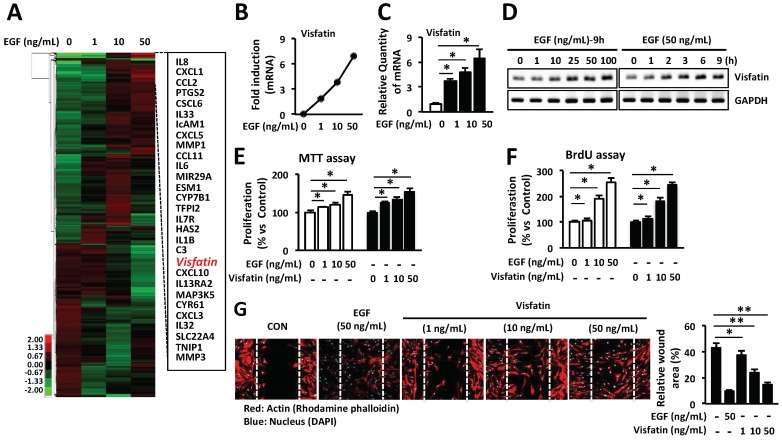
Visfatin stimulates the proliferation and migration of HDFs. (**A**) Hierarchical clustering of genes that were more than 2-fold differentially expressed in cDNA microarray. (**B**) HDFs were seeded into 6-well plates overnight and then cultured in a serum-free medium for further 24 h. Cells were treated with EGF or phosphate-buffered saline (PBS) at various concentrations and time points. The expression of visfatin mRNA was determined by cDNA microarray, (**C**) Q-PCR; * *p* < 0.05 compared to the saline-treated control group. Values represent the mean ± S.D. (*n* = 6) and (**D**) RT-PCR. Data are representative of three independent experiments. (**E**) HDFs were seeded in 96-well plates overnight and cultured in a serum-free medium for further 24 h. Cells were then treated with visfatin, EGF, or PBS at the indicated concentrations for 24 h. The proliferation of HDFs was determined by MTT assay (**E**) and BrdU cell proliferation ELISA assays (**F**) as described in the Materials and Methods section. * *p* < 0.05 compared to the saline-treated control group. Values represent the mean ± S.D. (*n* = 6). (**G**) HDFs were scratched with a 200-µL tip and treated with visfatin, EGF, or PBS at various concentrations for 24 h. Subsequently, cells were fixed with 4% paraformaldehyde for 10 min and stained with rhodamine phalloidin (red) and 4′6-diamidino-2-phenylindole (DAPI) (blue) for actin and nucleus staining, respectively. * *p* < 0.05, ** *p* < 0.01 compared to the control group. Values represent the mean ± S.D. (*n* = 6). Data are representative of three independent experiments.

The proliferation and migration of skin dermal fibroblasts play an important role in wound repair. HDFs express numerous genes in response to epidermal growth factor (EGF) stimulus. We hypothesized that EGF-induced genes are directly or indirectly involved in the enhancement of cell proliferation and migration in wound repair. Thus, in this study, we performed a cDNA microarray to characterize the wound-related genes and investigate the role of these genes in wound healing in vitro and in vivo. According to cDNA microarray analysis, out of a total number of genes analyzed (36,079), expression of 1210 genes (3.35%) was up- or down-regulated more than 2-fold in EGF-stimulated HDFs, compared with the untreated control. As shown in Figure 1A, as previous studies reported, we also found the up-regulated wound healing-related genes such as prostaglandin synthase 2 (*PTGS2*), intracellular adhesion molecule 1 (*ICAM-1*), matrix metalloproteinase 1 (*MMP1*), *MMP3*, hyaluronan synthase 2 (*HAS2*), and chemokines including interleukin-1 (*IL-1*), chemokine (C-X-C motif) ligand-1 (*CXCL-1*), chemokine (C-C motif) ligand 2 (*CCL2*), *CXCL-5*, *CCL11* and *CXCL10* in EGF-stimulated HDFs. Notably, EGF markedly increased the expression of visfatin in a dose-dependent manner in HDFs (Figure 1A,B). Based on several previous studies suggesting that visfatin promotes cell proliferation, migration and angiogenesis [26,27,28,29], we have selected a gene that visfatin could be closely related to wound healing. To further confirm the induction of visfatin expression, the expression levels of visfatin mRNA was determined by Q-PCR and RT-PCR. As shown in Figure 1C,D, we found that HDFs induced the expression of visfatin in a dose- and time-dependent manner in response to EGF stimulus.

We examined the effects of visfatin on the proliferation of HDFs by MTT (3-(4,5-dimethylthiazol-2-yl)-2,5-diphenyltetrazolium bromide) and BrdU (5-Bromo-2′-deoxyuridine) cell proliferation assays, the important steps of wound healing. As shown in Figure 1E, in cells treated with 50 ng/mL visfatin, the proliferation of HDFs was significantly increased by approximately 155.43 ± 7.94% compared to untreated control cells in MTT assay. The proliferation of visfatin-stimulated HDFs was comparable to that of EGF-treated HDFs (146.77 ± 6.72%) at 50 ng/mL concentration. Furthermore, as shown in Figure 1F, visfatin markedly increased cell proliferation in a dose-dependent manner, as demonstrated by the BrdU assay. The proliferation of visfatin-stimulated HDFs was comparable to that of EGF-stimulated HDFs (Figure 1F). Additionally, we observed that visfatin induced the migration of HDFs in a dose-dependent manner (Figure 1G). A confluent monolayer of HDFs was scratched with a 200-µL pipette tip and HDFs were stimulated for 24 h with various concentrations of visfatin. HDFs actively migrated into the scratched area at a concentration of 50 ng/mL visfatin, which was comparable with the migration of HDFs upon treatment with 50 ng/mL EGF. Thus, EGF-induced up-regulation of visfatin suggests the possibility that it plays an important role in wound repair.

### 2.2. Involvement of VEGF in Visfatin-Mediated Wound Healing

**Figure 2:**
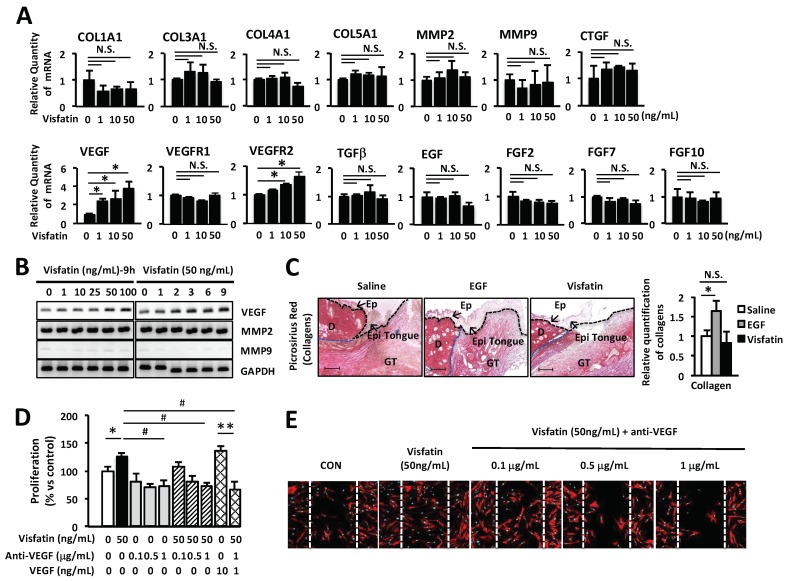
Visfatin-induced VEGF expression is involved in wound healing in HDFs. (**A**) HDFs were seeded into 6-well plates and then cultured in a serum-free medium for 24 h. Cells were treated with visfatin at various concentrations and time points. Expression of various genes was determined by Q-PCR (**A**,**B**) RT-PCR. * *p* < 0.05 compared to the saline-treated control group. Values represent the mean ± S.D. (*n* = 6). N.S.: not significant. (**C**) The wound tissues of day 6 post-wound were stained with picrosirius red for the production of collagens (Magnification 100×, Scale bar, 200 μm). The black arrow indicates as follow: D (dermis), Ep (epidermis), Epi tongue (epithelial tongue), and GT (granulation tissue). The production of collagens was measured and graphed. * *p* < 0.05 compared to the saline-treated control group. Values represent the mean ± S.D. (*n* = 6). N.S.: not significant. (**D**) HDFs were seeded into 96-well plates overnight and then cultured in a serum-free medium for 24 h. Cells were treated with visfatin or PBS at the indicated concentrations for 24 h after pretreatment with 0.1, 0.5, or 1 µg/mL VEGF-neutralizing antibody. The proliferation of HDFs was determined by MTT assay. * *p* < 0.05 compared to the saline-treated control group. ^#^ *p* < 0.01 compared to the visfatin-treated group. ** *p* < 0.01 compared to the VEGF-treated control group. Values represent the mean ± S.D. (*n* = 6). (**E**) HDFs were scratched with a 200-µL tip and subsequently treated with visfatin at various concentrations for 24 h after treatment with 0.1, 0.5, or 1 µg/mL VEGF-neutralizing antibody. Subsequently, cells were fixed with 4% paraformaldehyde and stained with rhodamine phalloidin (red) and DAPI (blue) for actin and nucleus staining, respectively. Data are representative of three independent experiments.

Several recent studies have suggested that visfatin might be closely related to the regulation of angiogenesis in various tumors, cell proliferation and migration [26,27,28,29]. Especially, VEGF plays a crucial role in cell proliferation and migration of keratinocytes and fibroblasts [32,37]. Here we examined the effects of visfatin on wound healing-related genes including collagens, *MMPs*, connective tissue growth factor (*CTGF*), EGF, fibroblast growth factors (FGFs), transforming growth factor beta (*TGFβ*), *VEGF*, VEGF receptor 1 (*VEGFR1*), and *VEGFR2* by Q-PCR analysis. We found that visfatin increased the expression of *VEGF* and *VEGFR2* (Figure 2A). However, visfatin did not affect the expression of other genes including MMPs, FGFs, CTGF, EGF and TGFβ. Furthermore, we confirmed the effects of visfatin on the expression of VEGF in HDFs. HDFs were cultured with various concentrations (0, 1, 10, 25, 50, and 100 ng/mL) of visfatin or EGF (positive control) for different time periods (0, 1, 2, 3, 6, and 9 h). The mRNA expression of VEGF was determined by RT-PCR. Visfatin up-regulated the expression of VEGF gene in a dose- and time-dependent manner (Figure 2B). However, visfatin did not affect the expression of matrix metalloproteinase 2 (MMP2) and MMP9, which also play an important role in the wound repair mechanism (Figure 2B). Moreover, visfatin did not induce the production of collagens in mouse skin wound, compared with the saline-treated skin wound as a control group (Figure 2C). In contrast, EGF-treated skin wound as a positive control exhibited significantly increased production of collagens, compared with the saline-treated mouse skin wound. To further determine the role of visfatin-induced VEGF expression in wound healing, HDFs were treated with visfatin in the presence of VEGF-neutralizing antibody. As shown in Figure 2D, the visfatin-induced proliferation of HDFs was inhibited by VEGF-neutralizing antibody in a dose-dependent manner. The proliferation of VEGF-treated HDFs was completely inhibited by VEGF-neutralizing antibody (Figure 2D). Furthermore, the VEGF-neutralizing antibody-treated HDFs showed significant inhibition of cell migration (Figure 2E). These results suggest that visfatin plays a critical role in wound healing through the induction of VEGF expression in HDFs.

### 2.3. Wound Healing Is Induced by Visfatin via ERK1/2 and JNK1/2 Activation in HDFs

**Figure 3:**
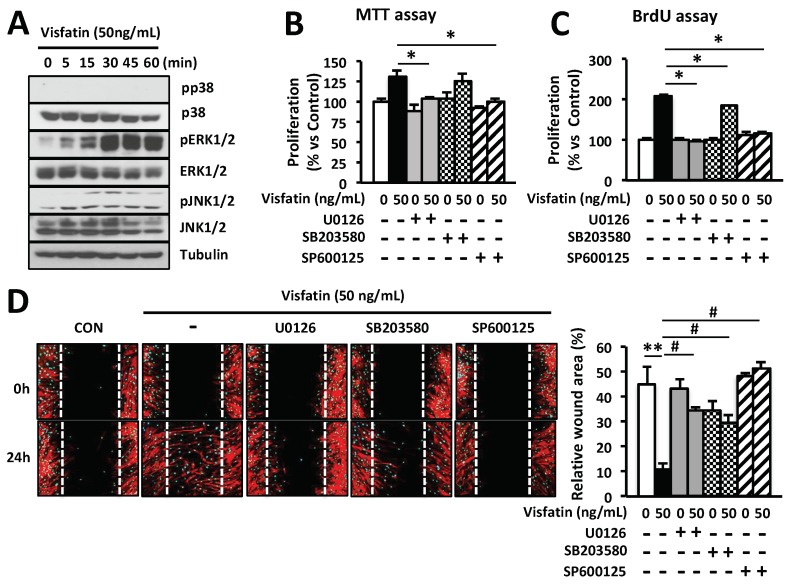
Visfatin enhances the proliferation and migration of HDFs through activation of ERK1/2 and JNK1/2. (**A**) HDFs were cultured for 24 h and then treated with 50 ng/mL visfatin for 5, 15, 30, 45 and 60 min. Cells were harvested and lysed and cell lysates were analyzed for the levels of p38, pp38, ERK1/2, pERK1/2, JNK1/2, and pJNK1/2 using the indicated antibodies; Tubulin was used as a loading control. (**B**,**C**) Cells were treated with 50 ng/mL visfatin for 24 h after pretreatment with 10 µM U0126, 20 µM SB203580, and 20 µM SP600125 for 1 h. The growth of HDFs was determined by MTT (**B**) and BrdU proliferation assays (**C**). * *p* < 0.05 compared to the visfatin-treated group. Values represent the mean ± S.D. (*n* = 6). (**D**) HDFs were scratched with a 200-µL tip and subsequently treated with visfatin for 24 h after pretreatment with 10 µM U0126, 20 µM SB203580, and 20 µM SP600125 for 1 h. Subsequently, cells were fixed with 4% paraformaldehyde and stained with rhodamine phalloidin (red) and DAPI (blue) for actin and nucleus staining, respectively. ** *p* < 0.01 compared to the control group. ^#^ *p* < 0.01 compared to the visfatin-treated group. Values represent the mean ± S.D. (*n* = 6). Data are representative of three independent experiments.

MAP kinase-signaling pathway has been shown to be involved in the proliferation and migration of skin keratinocytes and fibroblasts [42,43,44]. To investigate the effects of visfatin on the activation of MAP kinases in HDFs, cells were treated with visfatin and phosphorylation of each MAP kinase was determined by western blot analysis. As shown in Figure 3A, visfatin rapidly phosphorylated ERK1/2 and c-Jun N-terminal kinase (JNK1/2) within 5 min. This phosphorylation reached a maximum 30 min after stimulation with visfatin and was maintained until 60 min. Visfatin-induced phosphorylation of JNK1/2 was in a time-dependent manner which was subsequently decreased (Figure 3A). However, we observed that visfatin did not affect phosphorylation of p38 MAP kinase in HDFs.

To further investigate the role of ERK1/2, p38, and JNK1/2 phosphorylation in visfatin-induced proliferation and migration, HDFs were treated with U0126, SB203580, and SP600125, which are specific inhibitors of ERK1/2, p38, and JNK1/2, respectively. As shown in Figure 3B–D, visfatin-induced proliferation and migration of HDFs were inhibited by the specific inhibitors of ERK1/2 and JNK1/2. Taken together, these results indicate that visfatin induces the proliferation and migration of HDFs via activation of ERK1/2 and JNK1/2.

### 2.4. Wound Healing Activity Induced by Visfatin in Keratinocytes

**Figure 4:**
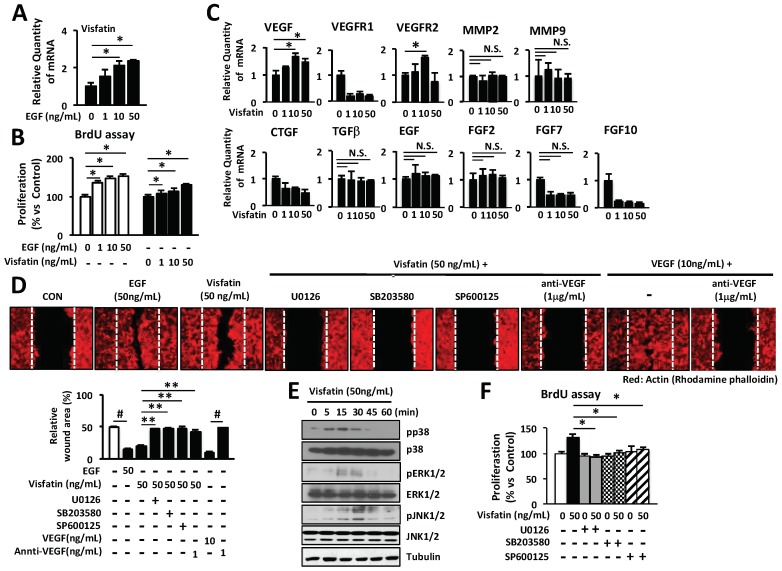
Visfatin enhances the proliferation and migration of human keratinocytes through activation of ERK1/2 and JNK1/2. (**A**) Human keratinocytes were seeded into 6-well plates overnight and then cultured in a serum-free medium for 24 h. Cells were treated with EGF at various concentrations or PBS as a negative control. The expression of visfatin mRNA was determined by Q-PCR. * *p* < 0.05 compared to the saline-treated control group. Values represent the mean ± S.D. (*n* = 6). Data are representative of at least three independent experiments. (**B**) Human keratinocytes were treated with visfatin, or EGF at the indicated concentrations for 24 h. The proliferation of keratinocytes was determined by BrdU cell proliferation assay. * *p* < 0.05 compared to the saline-treated control group. Values represent the mean ± S.D. (*n* = 6). (**C**) Human keratinocytes were treated with visfatin at various concentrations or PBS as a negative control. Expression of various genes was determined by Q-PCR. * *p* < 0.05 compared to the saline-treated control group. Values represent the mean ± S.D. (*n* = 6). N.S.: not significant. (**D**) Human keratinocytes were scratched with a 200-µL tip and treated with visfatin, EGF, or VEGF at the indicated concentration after pretreatment with 10 µM U0126, 20 µM SB203580, or 20 µM SP600125 for 1 h. Also, cells were treated with 50 ng/mL visfatin for 24 h after treatment with 1 µg/mL VEGF-neutralizing antibody. Subsequently, cells were fixed with 4% paraformaldehyde for 10 min and stained with rhodamine phalloidin (red) for actin. ^#^ *p* < 0.01 compared to the control- or VEGF-treated group. ** *p* < 0.01 compared to the visfatin-treated control group. Values represent the mean ± S.D. (*n* = 3). (**E**) Human keratinocytes were treated with 50 ng/mL visfatin for 5, 15, 30, 45, and 60 min. Cells were harvested and lysed and cell lysates were analyzed for the levels of p38, pp38, ERK1/2, pERK1/2, JNK1/2, and pJNK1/2 using the indicated antibodies. Tubulin was used as a loading control. (**F**) Human keratinocytes were treated with 50 ng/mL visfatin for 24 h after pretreatment with 10 µM U0126, 20 µM SB203580, and 20 µM SP600125 for 1 h. The cell growth of keratinocytes was determined by BrdU proliferation assay. * *p* < 0.05 compared to the visfatin-treated control group. Values represent the mean ± S.D. (*n* = 6). Data are representative of three independent experiments.

To further confirm the induction of visfatin expression by EGF treatment, the expression level of visfatin mRNA was determined in human keratinocytes by Q-PCR. As shown in Figure 4A, we found that keratinocytes in response to EGF stimulus induced the expression of visfatin in an EGF dose-dependent manner. Besides, we examined the effects of visfatin on the proliferation of keratinocytes by the BrdU cell proliferation assay. As shown in Figure 4B, the proliferation of keratinocytes was significantly increased by visfatin stimulation, compared to that of the untreated control cells. The proliferation of visfatin-stimulated keratinocytes was comparable to that of EGF-treated keratinocytes (Figure 4B). Also, we examined the effects of visfatin on the expression of the wound healing-related genes including collagens, MMPs, CTGF, EGF, FGFs, TGFβ, VEGF, VEGFR1 and VEGFR2 in human keratinocytes using Q-PCR. As shown in Figure 2A, we found that visfatin significantly increased the expression of VEGF and VEGFR2 (Figure 4C). However, visfatin did not affect the expression of other genes including MMPs, FGFs, CTGF, EGF, and TGFβ. Additionally, we observed that visfatin induced the migration of keratinocytes (Figure 4D). Keratinocytes actively migrated into the scratched area at a concentration of 50 ng/mL visfatin, which was comparable with the migration of keratinocytes upon treatment with 50 ng/mL EGF or 10 ng/mL VEGF. Importantly, VEGF-neutralizing antibody suppressed the visfatin-induced cell migration of keratinocytes (Figure 4D). Also, visfatin-induced migration of keratinocytes was completely inhibited by the specific inhibitors of p38, ERK1/2, and JNK1/2 (Figure 4D).

To investigate the effects of visfatin on the activation of MAP kinases in keratinocytes, cells were treated with visfatin and phosphorylation of each MAP kinase was determined by western blot analysis. As shown in Figure 4E, visfatin rapidly induced phosphorylation of p38, ERK1/2 and JNK1/2 within 5 or 15 min. This phosphorylation reached a maximum at 30 min after stimulation with visfatin (Figure 4E). The phosphorylation levels of p38 MAP kinase in visfatin-treated keratinocytes were comparable to those of the visfatin-treated HDFs (Figure 3A). To further investigate the role of ERK1/2, p38, and JNK1/2 phosphorylation in the visfatin-induced cell proliferation, keratinocytes were treated with U0126, SB203580, and SP600125, which are specific inhibitors of ERK1/2, p38, and JNK1/2, respectively. As shown in Figure 4F, the visfatin-induced proliferation of keratinocytes was inhibited by the specific inhibitors of p38, ERK1/2 and JNK1/2 (Figure 4F). Taken together, these results indicate that visfatin also induces the proliferation of keratinocytes via activation of p38, ERK1/2, and JNK1/2.

### 2.5. Induction of Wound Healing by Visfatin In Vivo

**Figure 5:**
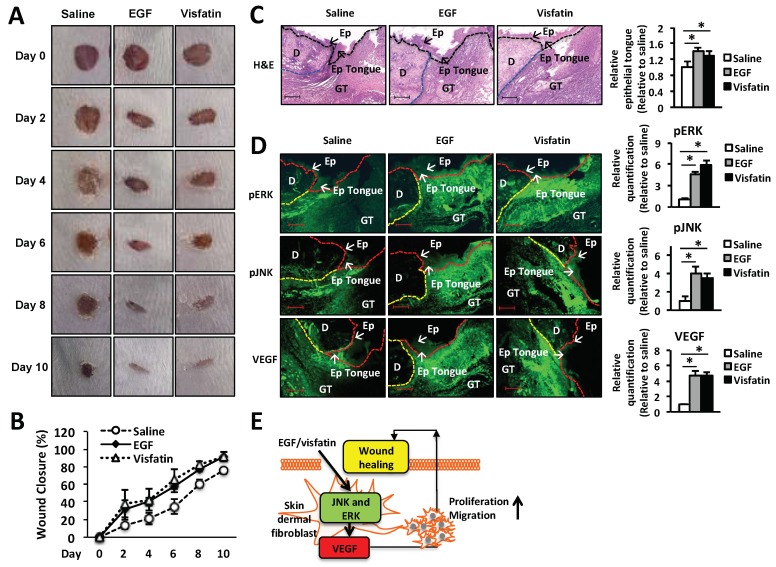
Treatment with visfatin accelerates wound healing in vivo. (**A**) Representative photographs of wounds from mice treated with visfatin, EGF, and control saline. The hydrogel containing visfatin (1 µg/wound) and EGF (1 µg/wound) were applied every 24 h for 5 days. Digital images indicate the status of wound healing during the 10 days of the wound repair process. (**B**) The wound closure in control saline-, visfatin-, or EGF-treated mice was measured digitally by counting pixels at the indicated days after surgical incision and the wound closure was expressed in terms of percentage. * *p* < 0.05 compared to the saline-treated control group. Values represent the mean ± S.D. (*n* = 6). (**C**) For the histological analysis, the H&E-stained wound tissues of day 6 post-wound were analyzed and graphed for epithelial tongue (Magnification 100×, Scale bar, 200 μm). The black arrow indicates as follow: D (dermis), Ep (epidermis), Epi tongue (epithelial tongue), and GT (granulation tissue). * *p* < 0.05 compared to the saline-treated control group. Values represent the mean ± S.D. (*n* = 6). N.S.: not significant. (**D**) For the immunofluorescence staining, tissue sections of day 6 post-wound were treated with fluorescence-conjugated antibodies against ERK, JNK, or VEGF and photographed (Magnification ×200, Scale bar, 200 μm). * *p* < 0.05 compared to the saline-treated control group. Values represent the mean ± S.D. (*n* = 6). (**E**) Proposed scheme of the therapeutic action of visfatin in wound healing.

To examine the therapeutic effects of visfatin on wound repair, 25 µL of 22% hydrogel containing 1 µg visfatin was applied to a 5-mm wound on the back of BALB/c mice once a day for 5 days. EGF (1 µg/wound) and vehicle alone (saline) were used as a positive and negative control, respectively. Wound repair was monitored every day for 10 days after injury and wound closure was analyzed by ImageJ for the percentage of wound size (Figure 5B). As shown in Figure 5A, in the hydrogel containing visfatin or EGF-treated mice the wound size was decreased significantly from day 2 to 10 after injury, compared to those treated with vehicle alone. Ten days after injury, the wounds of visfatin-treated mice were healed approximately 100% compared to those wounded and left (Figure 5B).

Next, the wound tissues were collected at day 6 after wound puncture and the histological analysis of the wound tissues were performed using hematoxylin and eosin (H&E) staining (Figure 5C). We found that visfatin-treated group increased the length of epithelial tongues and epidermis around the wound edge (Figure 5C). These results demonstrated that the visfatin-treated group restored the skin wound tissues, as similar to the EGF-treated group (Figure 5C). We further confirmed the mechanism by which visfatin activates ERK and JNK pathway in vivo. As shown in Figure 5D, the induction of phosphorylation of ERK and JNK was confirmed by the immunofluorescence staining of the tissue sections, compared to the saline-treated group (Figure 5D). The induction of ERK and JNK phosphorylation was comparable to that of the EGF-treated group (Figure 5D). Moreover, we demonstrated that visfatin increased the VEGF expression in the wound tissue section, compared to the saline-treated group. These results indicate that visfatin promotes wound healing in vivo. The therapeutic effect of visfatin on wound repair was comparable to that of EGF treatment.

## 3. Discussion

The clinical applications of growth factors, including epidermal growth factor (EGF) which is commercially available and basic fibroblast growth factor (bFGF) have been rapidly developed in wound healing. The topical applications of these growth factors have been proven to be effective in wound healing in China [45]. However, the mechanism by which EGF promotes wound healing is still unclear. Thus, identification of the EGF-induced genes might be helpful in understanding the process of wound healing. In this study, we have identified the wound healing-related gene, visfatin, by cDNA microarray analysis in EGF-stimulated human dermal fibroblasts (HDFs) and then examined the effects of visfatin on wound healing in vitro and in vivo. Many studies have reported that visfatin is secreted from peripheral-blood monocytes, lymphocytes, dendritic cells, macrophages, adipose tissues and expressed in various malignant tumors [13,15,17,18,19,20,21,22,23,24,46]. Additionally, visfatin was shown to be expressed in fibroblasts or macrophages in the synovial tissues with rheumatoid arthritis and psoriatic skin [47,48]. The function of visfatin has been examined in atherosclerotic diseases and various types of tumors, such as glioblastoma, malignant astrocytomas, breast and, prostate cancer [20,49,50,51,52,53]. Visfatin is also known to function as a growth factor causing proliferation and differentiation. Moreover, visfatin has been shown to promote vascular smooth muscle cell maturation and is well characterized for insulin mimetic effect [17,54]. However, there is no evidence to date on therapeutic effects of visfatin in wound repair. In this study, we have demonstrated the role of visfatin and its underlying mechanism in wound healing.

EGF significantly induced the expression of visfatin in a dose- and time-dependent manner in HDFs (Figure 1A–D). Thus, we hypothesized that visfatin could be involved in the wound healing process. As we expected, human recombinant visfatin significantly induced the migration and proliferation of HDFs (Figure 1E–G). However, the mechanism by which visfatin promotes wound repair is still unclear. The recent study demonstrated that visfatin induced the proliferation of endothelial cells by induction of VEGF production [27,39]. Visfatin was also reported to induce the expressions of MMP-2 and MMP-9 [27,39]. As shown in Figure 2, visfatin induced the expression of VEGF. However, MMP2 and MMP9 expression remained unchanged upon visfatin treatment (Figure 2A,B). We expect that this discrepancy depends on cell types used for the experiments. Also, we demonstrated that visfatin enhanced the expression of VEGF in wound tissue section (Figure 5D). Recent studies have reported that visfatin increases VEGF expression through activation of PI3K/Akt, ERK1/2, and p38 MAPK signaling pathways [26,27,39,40,41]. Consistent with these reports, we found that visfatin activated the phosphorylation of ERK1/2 and JNK1/2 MAPK in HDFs (Figure 3A). Furthermore, in the histological analysis, we found that visfatin increased the length of epithelial tongues and epidermis around the wound edge, as similar to the EGF-treated group (Figure 5C). As shown in Figure 5D, visfatin induced the phosphorylation of ERK and JNK in tissue sections, compared to the saline-treated group (Figure 5D). In addition, visfatin-induced proliferation and migration of HDFs were significantly suppressed by specific inhibitors of ERK1/2 and JNK1/2, that is, U0126 and SP600125, respectively (Figure 3B–D). We also found that visfatin improved wound healing by enhancing the proliferation and migration through the induction of VEGF via the MAPK pathway in keratinocytes, as similar to the HDFs model (Figure 4). Therefore, we demonstrated that visfatin induced the proliferation and migration of cells through ERK1/2 and JNK1/2 signaling pathways. As shown in Figure 5E, we suggested that visfatin promotes wound repair by enhancing VEGF expression via phosphorylation of the MAP ERK1/2 and JNK1/2.

The results of the present study have several critical clinical implications. First, EGF, a typical growth factor, significantly increased the expression of visfatin which was shown to play an important role in wound healing in a time- and dose-dependent manner in HDFs. Thus, we suggest that visfatin may be used as a novel therapeutic agent for wound repair along with other growth factors, such as EGF and bFGFs. Second, undefined receptors of visfatin should be demonstrated to improve the healing potential of visfatin in promoting wound healing. These data indicate that visfatin may become a potential therapeutic agent to improve tissue repair through triggering the proliferation and migration of HDFs and human keratinocytes via induction of VEGF expression. However, further studies on visfatin expression and activity are needed to develop a novel therapeutic agent for tissue repair.

## 4. Materials and Methods

### 4.1. Mice and Cell Culture

BALB/c mice were purchased from Orient Bio, Inc. (Seoul, Korea). All experiments were ethically performed under the guidelines of the Korea University Institutional Animal Care and Use Committee (Seoul, Korea; approval no. KUIACUC-2015-244, 2017-109). Human dermal fibroblasts (HDFs) and human keratinocytes (HaCaT cells) were purchased from Biosolution (Seoul, Korea) with passage number 2. HDFs and HaCaT cells were cultured in the mixture (1:3) of F-12K and DMEM or DMEM with 10% fetal bovine serum and 100 U/mL penicillin and 0.1 mg/mL streptomycin (Life Technologies, Carlsbad, CA, USA) at 5% CO_2_, 37 °C incubator, respectively.

### 4.2. Reagents and Antibody

Antibodies for JNK1/2 (sc-571), phosphor-JNK1/2 (sc-6254), ERK1/2 (sc-153), phosphor-ERK1/2 (sc-7383), p38 (sc-535), Tubulin (sc-69969), and VEGF (sc-152) were purchased from Santa Cruz Biotechnology (Dallas, TX, USA). Antibodies for goat anti-rabbit IgG Alexa fluor 488 (A-11034) and goat anti-mouse IgG Alexa fluor 488 (A-11029) secondary antibody were purchased from Invitrogen (Carlsbad, CA, USA). Antibodies for phospho-p38 (#9216), Rabbit-HRP (#7074) and Mouse-HRP (#7076) were purchased from Cell Signaling (Danvers, MA, USA). Recombinant human visfatin (10990-H20B) was purchased from Sino Biological (Beijing, China). Pluronic^®^F-127 (P2443) for the preparation of hydrogel (22%) to delivered EGF or visfatin was purchased from Sigma Aldrich (Kenilworth, Merck, Germany).

### 4.3. cDNA Microarray

Fragmented and Labeled single strand-DNA (ss-DNA) was prepared according to the standard Affymetrix protocol from 500 ng total RNA (GeneChip^®^ WT PLUS Reagent Kit Manual, Carlsbad, CA, USA). Following fragmentation, 3.5 µg of ss-DNA were hybridized for 16 h at 45 °C with 60 rpm on GeneChip^®^ Mouse Gene 2.0 ST Array (Carlsbad, CA, USA). GeneChips were washed and stained on the Affymetrix Fluidics Station 450 (Carlsbad, CA, USA). GeneChips were scanned using the Affymetrix GeneChip Scanner 3000 7G (Carlsbad, CA, USA). To normalize the results, the data were analyzed with Robust Multichip Analysis (RMA) using Affymetrix default analysis settings and global scaling. The trimmed mean target intensity of each array was arbitrarily set to 100. The values of normalized and log-transformed intensity were then analyzed using GeneSpring GX 13.1.1 (Agilent Technologies, Santa Clara, CA, USA). Fold change filters included the requirement that the genes be present in at least 200% of controls for up-regulated genes (2-fold up) and lower than 50% of controls for down-regulated genes (2-fold down).

### 4.4. MTT and BrdU Proliferation Assays In Vitro

HDFs and HaCaT cells were seeded and pre-cultured into 96-well plates at a density of 5 × 10^3^ cells/well for 24 h. After cells were cultured in serum-free media for 16 h and then cells were stimulated with various concentration of visfatin or EGF as a positive control in serum-free medium. After 24 h stimulation, the cells were treated with 20 µL of 100 µg/mL of 3-(4,5-Dimethylthiazol-2-yl)-2,5-diphenyltetrazolium bromide (MTT) solution for 2 h and then dissolved in 150 µL of DMSO and then determined absorbance at 560 nm. For BrdU incorporation assay, the 5-bromo-2′-deoxy-uridine (BrdU) cell proliferation ELISA kit (colorimetric) was purchased from Roche (11647229001, Basel, Switzerland) and the assay was performed according to the manufacturer’s instructions. In brief, after 24 h stimulation with visfatin or EGF, cells were treated with BrdU for 3 h. BrdU-integrated DNA was determined at 490 nm and quantified according to the relative absorbance.

### 4.5. Migration Assay In Vitro

HDFs and HaCaT cells were seeded and pre-cultured into 12-well plates at a density of 8 × 10^4^ cells/well for 24 h and then cells were cultured with serum-free media for 16 h. HDFs were scratched with the 200-µL tip. Cells were treated with a various concentration of visfatin or EGF for 24 h. Following stimulation, the cells were fixed with 4% paraformaldehyde for 10 min at room temperature and then permeabilized with 0.1% Triton X-100 in PBS for 5 min. Next, the cells were stained with rhodamine phalloidin for 2 h. Nuclei were stained with DAPI. Fluorescence images were acquired with a fluorescence microscope (Olympus IX 71, Shinjuku, Tokyo, Japan).

### 4.6. Wound Healing Assay In Vivo

Seven-week-old mice (*n* = 6) were anesthetized with 65% N_2_, 30% O_2_ and 5% isoflurane and then maintained with 2% isoflurane during wound puncture. Dorsal hair was shaved and wiped with 70% ethanol before wound puncture (5 mm diameter). The wounds were topically treated with saline, 1 μg of EGF, or 1 μg of visfatin in hydrogel per wound at once a day for 5 days. The wound closure was monitored by taking the picture at the same height with fixed height stand. Measurement of the wound area in the images was analyzed using by the Image J program (NIH, Bethesda, MD, USA). Wound repair was calculated using the percentage of wound size as follows [55]:

$$Wound\;closure\;\left(\%\right)=\frac{\left(Wound\;Area\;on\;Day\;0\right)-\left(Wound\;Area\;on\;Day\;post-wounding\right)}{Wound\;Area\;on\;Day\;0}\times 100$$

### 4.7. Histologic Analysis of Wounds and Immunofluorescence Microscopy

Histologic analysis was performed on digital images using the ImageJ program. Cross-sections (5 µM) of paraffin-embedded skin tissues of day 6 post-wound were stained with hematoxylin and eosin (H&E) or picrosirius red for the extent of re-epithelialization and collagen expression, respectively. Measurement of the collagen in the images was analyzed using by the ImageJ program. The length of the epithelial tongue was determined as the distance between the margin of the wound and the end of the epithelial using by ImageJ program. The relative epithelial tongue was calculated compared with the saline-treated group. For immunofluorescence microscopy, immunofluorescence detection of phospho-ERK, phosphor-JNK, and VEGF proteins was performed using rabbit anti-VEGF, anti-mouse pERK, anti-mouse pJNK (Santa Cruz Biotechnology; Dallas, TX, USA), goat anti-rabbit IgG Alexa fluor 488 (A-11034), and goat anti-mouse IgG Alexa fluor 488 (A-11029) secondary antibody (Invitrogen, Carlsbad, CA, USA) in the paraffin section of mouse skin tissue of day 6 post-wound. Stained sections were observed under fluorescence microscopy systems (100×, Olympus IX 71, Shinjuku, Tokyo, Japan). The fluorescence was analyzed and quantified using by the ImageJ program.

### 4.8. Reverse Transcription-Polymerase Chain Reaction (RT-PCR) and Real-Time Quantitative PCR (Q-PCR) Analysis

HDFs (1 × 10^5^ cells/well) were treated with various concentrations of visfatin for the different time and then total RNA was extracted with RiboEX total RNA kit (GeneAll Biotechnology, Seoul, Korea). Total RNA (1 μg) was reverse-transcribed into cDNA by reverse transcriptase. The cDNAs were amplified with specific primers by PCR using GenieTM 32 Thermal Block (Bioneer, Daejeon, Korea). Specific primer sequences were as follow: hGAPDH, 5′-ACA TCA AGA AGG TGG TGA AG-3′ (sense) and 5′-ATT CAA GAG AGT AGG GAG GG-3′ (anti-sense); hvisfatin, 5′-AGG GTT ACA AGT TGC TGC CA-3′ (sense) and 5′-CAA AAT TCC CTG CTG GCG TC-3′ (anti-sense); hVEGF, 5′-CCT TGC CTT GCT GCT CTA CC-3′ (sense) and 5′-CCT ATG TGC TGG CCT TGG TG-3′ (anti-sense). The PCR products were separated on 1.5% agarose gels and stained with Staining Star (Dyenbio, Seongnam, Kyeongki, Korea). Quantitative-PCR analyses were performed with Fast SYBR^TM^ Green Master Mix (Applied Biosystems^TM^, Waltham, MA, USA). The reactions of 6.25 µL fast SYBR^TM^ Green Master Mix, 1 μL of cDNA, 0.5 µM primers in a final volume of 12.5 μL were analyzed in an optical 96-well fast clear reaction plate (Applied Biosystems^TM^, Waltham, MA, USA). Reactions were amplified and using human GAPDH as an endogenous control. The primer sequences are listed in quantified by using a StepOnePlus Real-Time PCR System and the manufacturer’s corresponding software (StepOnePlus Software v2.3; Life Technologies, Waltham, MA, USA). The relative quantities of mRNAs were determined by using the comparative Ct method and were normalized Table 1.

### 4.9. Western Blot Analysis

Whole cell lysates were separated on 10% SDS–PAGE gels and transferred to polyvinylidene difluoride membranes (PVDF). The membrane was blocked with 5% skim milk (Neogen Co., Lansing, MI, USA) and then incubated with a 1:1000 diluent of a primary antibody in TBS with 5% BSA. The membrane was washed 5 times in TBS-T and then incubated with 1:5000 diluent of the corresponding secondary antibodies HRP-conjugated IgG antibody (Cell Signaling Technology, Danvers, MA, USA) in TBS with 5% skim milk. Target proteins were visualized by using the ECL detection system (Amersham ECL Prime Western blotting Detection Regent, GE Healthcare, Chicago, IL, USA).

### 4.10. Statistical Analysis

All experiments were repeated at least three independent experiments. Data in bar graphs were shown as the mean ± standard deviation (S.D.). Statistical analysis between the control and the treated groups were assessed using a two-tailed Student’s *t*-test, Normality of distribution was assessed by the Shapiro-Wilk test and comparisons more than two conditions (k > 2) were determined using one-way analysis of variance (ANOVA) followed by Tukey post-hoc test, using SPSS 22 statistics software (IBM). * *p* < 0.05, ** *p* < 0.01 and ^#^ *p* < 0.01 were considered statistically significant.

## Figures and Tables

**Table 1:** Primer sequences used for Q-PCR.

Gene	Forward (5′-3′)	Reverse (5′-3′)
hCOL1A1	CATGACCGAGACGTGTGGAA	GGCAGTTCTTGGTCTCGTCA
hCOL3A1	TGTTCCAGGAGCTAAAGGCG	CTCCTGGGATGCCATTTGGT
hCOL4A1	TTTTGTGATGCACACCAGCG	AGTAATTGCAGGTCCCACGG
hCOL5A1	AGACATGGGCATCAAGGGTG	CCGAGTTTTCCCTTCTCCCC
hMMP2	CCCTGTGTCTTCCCCTTCAC	GTAGTTGGCTGTGGTCG
hMMP9	GGACAAGCTCTTCGGCTTCT	TCGCTGGTACAGGTCGAGTA
hFGF2	AAAAACGGGGGCTTCTTCCT	AGCCAGGTAACGGTTAGCAC
hFGF7	TGGATCCTGCCAACTTTGCT	TTCTTGTGTGTCGCTCAGGG
hFGF10	GGAGCTACAATCACCT	ACGGGCAGTTCTCCTTCTTG
hEGF	AGTCCGTGACTTGCAAGAGG	CCTCTTCTTCCCTAGCCCCT
hTGFβ	TGGTGGAAACCCACAACGAA	GAGCAACACGGGTTCAGGTA
hCTGF	GTTTGGCCCAGACCCAACTA	GGCTCTGCTTCTCTAGCCTG
hVEGF	CTTGCCTTGCTGCTCTACCT	GCAGTAGCTGCGCTGATAGA
hVisfatin	TCGGTTCTGGTGGAGGTTTG	TTGGGATCAGCAACTGGGTC
hVEGFR1	GGGGGAAGCAGCCCATAAAT	GCCAGTGTGGTTTGCTTGAG
hVEGFR2	CGGTCAACAAAGTCGGGAGA	CAGTGCACCACAAAGACACG
hGAPDH	TCCTGCACCACCAACTGTT	GTCCACTGTCTTCTGGGTGG
